# Hyperinsulinemia/euglycemia and intravenous lipid emulsion therapy for the management of severe amlodipine toxicosis in a cat

**DOI:** 10.1002/ccr3.5175

**Published:** 2021-12-07

**Authors:** Audrey E. Tinsman, Tara J. Bellis

**Affiliations:** ^1^ BluePearl Specialty + Emergency Pet Hospital New York NY USA; ^2^ Emergency & Critical Care Garden State Veterinary Services Iselin NJ USA

**Keywords:** amlodipine toxicosis, feline, hyperinsulinemia, intralipid therapy, refractory hypotension

## Abstract

Calcium‐channel blockers (CCBs) are widely used in people and animals. Overdose can result in cardiovascular collapse and death. Hyperinsulinemia/euglycemia therapy (HIET) and intralipid therapy (ILT) are reported treatment options in people. This is the first report describing HIET and ILT as treatments for amlodipine toxicosis in a cat.

## Graphical abstract

Hyperinsulinemia/euglycemia therapy (HIET) and intralipid therapy (ILT) should be considered for the treatment of cardiac dysfunction and refractory hypotension secondary to amlodipine toxicosis in cats.

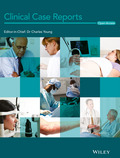

## 1 INTRODUCTION

Calcium‐channel blockers (CCBs) have been used for over 40 years in veterinary medicine as both anti‐hypertensive and anti‐arrhythmic medications.^1^ Three main categories of CCBs are commercially available in the United States for veterinary use: phenylalkylamines, such as verapamil; benzothiazepines, such as diltiazem; and dihydropyridines, such as amlodipine. All are defined by their ability to block the long‐lasting L‐type calcium channels found primarily in the cardiac, smooth, and skeletal muscle.^1^ Calcium channels are vital for conduction of electrical impulses across the sinoatrial and atrioventricular nodes of the heart. By inhibiting calcium influx, CCBs decrease atrioventricular conduction and slow the heart rate. In contractile cells, such as vascular smooth muscle cells and cardiac myocytes, calcium‐channel blockade reduces cytosolic calcium levels and calcium‐induced calcium release from the sarcoplasmic reticulum.^2^ This leads to reduced cardiac inotropy and vasodilation, particularly in vascular beds with a high‐resting tone. Arterioles are the primary modulatory vessels of systemic vascular resistance (SVR) and have a dose‐dependent response to CCBs.^3^ Other tissues affected by CCBs include the pancreas, pulmonary parenchyma, and central nervous system.^1^ Blockade of L‐type calcium channels in the pancreas causes decreased insulin release by the β‐islet cells and restricts tissue uptake of glucose by altering sensitivity to insulin.^1^ Under normal aerobic conditions, myocardial cells oxidize free fatty acids as their main energy substrate. In hypoperfused or hypoxic states, myocardial cells undergo a metabolic shift to utilize glucose as their primary substrate. In patients with CCB toxicosis, hypoinsulinemia, insulin resistance, and hypotension lead to reduced glucose delivery and uptake by the cells. That combined with an increased tissue demand causes a relative negative glucose balance leading to impaired inotropy and myocardial dysfunction.^4^

The most commonly reported clinical signs of CCB toxicity are exaggerated therapeutic effects, namely hypotension, bradycardia, and bradyarrhythmias. Hyperglycemia is observed due to altered pancreatic function as previously discussed. Other symptoms of hypoperfusion such as hypothermia and depression are reported. Electrolyte disturbances including hypokalemia and hyponatremia are common, and a metabolic acidosis in conjunction with hyperlactatemia is of note. Non‐cardiogenic pulmonary edema has also been reported in people.^23^ Stimulatory signs of the central nervous system such as agitation or seizures have been observed but are considered rare.^1^ First‐line treatments for CCB toxicosis include decontamination and supportive care; second‐line treatments include calcium supplementation, insulin‐glucose infusions, vasopressors/catecholamines, glucagon, intralipid therapy, and external pacing.^1^

Hyperinsulinemia/euglycemia therapy (HIET) is well described in human medical literature for the treatment of CCB toxicity; however, it is infrequently reported in veterinary medical literature. It consists of a high‐dose regular insulin infusion coupled with a glucose infusion.^6^ While the ideal dose has not been established in veterinary medicine, a widely accepted human protocol consists of an initial intravenous insulin bolus of 1 U/kg, followed by a continuous rate infusion (CRI) of 0.5–2 U/kg/h in conjunction with 10% dextrose solution IV. If clinical signs remain refractory to treatment, the insulin dose can be titrated up to 10 U/kg/h and dextrose adjusted accordingly. Blood glucose monitoring is recommended every 5–30 min.^1^ Intracellular transport of glucose in cardiac and skeletal muscle is greatly enhanced by insulin providing an ongoing source of energy for cardiac contraction. In high concentrations, insulin affects several intracellular pathways that increase the inotropy of cardiac myocytes and alter calcium handling.^2^ Additional benefits of HIET include increased cyclic AMP levels through phosphodiesterase III inhibition, resulting in increased calcium influx.^6^ There is evidence that HIET increases endothelial nitric oxide synthase (eNOS) activity which in turn decreases capillary resistance and improves overall tissue perfusion.^5^ There is one published case report in the veterinary literature describing HIET for the treatment of CCB toxicosis in a dog. Maton et al describe the successful use of HIET in the treatment of diltiazem toxicosis.^7^ To the authors’ knowledge, this is the first case report describing the use of HIET for the treatment of CCB toxicosis in a cat.

## 2 CASE SUMMARY

A 15‐year‐old female patient spayed Siamese cat weighing 3.19 kg presented for amlodipine toxicosis. Two months prior the cat was treated by her primary veterinarian for a urinary tract infection and diagnosed with systemic hypertension. Antihypertensive therapy with telmisartan 6.5 mg PO q24h (Semintra, Boehringer Ingelheim Vetmedica, Inc.) was initiated after repeatable blood pressure measurements greater than 190 mm Hg were obtained at the referring veterinarian. The patient was transitioned onto amlodipine two weeks prior to hospitalization due to the availability and cost‐constraints of telmisartan. For the five days leading up to presentation, the cat received 2.5 mg instead of 0.625 mg PO q24h. The cat had known pre‐existing renal disease (IRIS stage 2), which was managed with a prescription diet and stool softener (Miralax, Bayer Healthcare LLC; 1/8tsp PO q12h).

The cat presented to the authors' hospital for decreased appetite and water intake for 24 h and lethargy for several days. The owner reported a single episode of vomiting the night before. Amlodipine was last administered 16 h prior to evaluation. Upon presentation (day one), the cat was dehydrated with a prolonged skin tent and weak femoral pulses. She had a decreased axillary temperature (35.5°C; 38.1–39.2°C)^8^ and mild bradycardia (160 beats per minute; 160–220 beats per minute).^10^ She was weakly ambulatory and quiet. Systolic blood pressure (SBP) as measured by Doppler ultrasonography (Doppler flow detector model 811‐B, Parks Medical Electronics) was low at 70 mm Hg (110–132 mm Hg).^9^ An intravenous (IV) catheter was placed, and a minimum database was performed, which identified mild anemia and hyperproteinemia (Table 1). Spot blood glucose (sBG), (AlphaTRAK 2, Abbott Animal Health) was high at 14.37 mmol/L (259 mg/dl; RI [3.72–9.32 mmol/L]).^10^ A venous blood gas (vBG; RAPIDPoint 500 System, Siemens Healthineers) revealed a metabolic acidosis with hyperlactatemia (Table 1). A chemistry panel (Catalyst One, IDEXX Laboratories Inc) revealed azotemia and hyperamylasemia (Table 2). A 10 mls/kg IV crystalloid bolus of LRS (Lactated Ringer's Solution; Dechra Veterinary Products) was administered. Systolic BP improved to 92 mm Hg. A 90 mg/kg calcium gluconate 10% (Calcium Gluconate Injection USP 100 mg per ml, HF Acquisition Co LLC, DBA HealthFirst) bolus diluted 1:1 with NaCl 0.9% (0.9% Sodium Chloride Injection USP, B. Braun Medical) was administered intravenously over 20 min. Maropitant citrate (Cerenia 10 mg/ml, Zoetis Inc; 1 mg/kg IV q24h) was administered, and the patient was hospitalized on isotonic fluid therapy NaCl 0.9% at 8 mls/h.

**TABLE 1:** Serial venous blood gas, electrolytes, PCV, and TP measurements

	0:00^a^	10:00^a^	13:00^a^	15:00^a^	19:00^a^	23:00^a^	27:00^a^	31:00^a^	Ref Range^25^
PCV (%)	25	24	25	24	24	24	24	22	25 to 45
TPP (g/dl)	9.0	6.4	10	10	8.6	9.4	6.4	6.0	60 to 86
pH	7.245	7.130	7.067	7.090	7.185	7.142	7.230	7.202	7.24 to 7.44
pCO_2_ (mm Hg)	30.7	39.6	43.9	39.0	30.6	35.6	32.5	34.5	27.3 to 49.1
HCO_3_ (mmol/L)	13.0	12.9	12.3	11.6	11.3	11.9	13.3	13.2	15.9 to 24.7
BE (mmol/L)	−13.1	−15.2	−17.5	−17.1	−15.6	−15.8	−13.1	−13.6	−9.5 to −0.3
Na (mmol/L)	143.1	140.8	142	142.6	148.2	145.0	144.2	146.4	150.5 −157.2
K (mmol/L)	3.55	3.75	2.59	2.82	2.85	3.04	4.53	4.99	3.11 to 4.64
Ca (mmol/L)	1.01	1.54	1.80	1.87	1.72	1.52	1.34	1.48	–
Cl (mmol/L)	118	116	119	121	122	125	124	125	113 to 123
Glu (mg/dl)	206	332	114	90	50	119	96	190	77 to 158
Lac (mmol/L)	3.73	2.75	1.65	1.32	1.00	1.23	0.72	0.87	0.61 to 5.86

^a^ Time since hospitalization.

**TABLE 2:** Complete blood cell count and chemistry values

	Value	Reference
Complete cell count		
Hematocrit (%)	23.3	30.3–52.3
WBC (K/Ul)	16.24	2.87–17.02
Neutrophils (K/Ul)	10.99	2.30–10.29
Monocytes (K/Ul)	1.34	0.05–0.67
Eosinophils (K/Ul)	0.49	0.17–1.57
Platelets (K/Ul)	298	151–600
Chemistry		
Glucose (mg/dl)	207	71–159
BUN (mg/dl)	73	16–36
Creatinine (mg/dl)	2.4	0.8–2.4
Total protein (G/dl)	6.8	5.7–8.9
Albumin (G/dl)	3.1	2.3–3.9
Globulin (G/dl)	3.7	2.8–5.2
Calcium (mg/dl)	8.5	7.8–11.3
Phosphorus (mg/dl)	6.3	3.1–7.5
ALT (U/L)	25	12–130
ALP (U/L)	24	14–111
Total bilirubin (mg/dl)	0.7	0.0–0.9
Cholesterol (mg/dl)	156	65–225
Amylase (U/L)	>2500	500–1500
Cholesterol (mg/dl)	156	65–225
Lipase (U/L)	966	100–1400
GGT (U/L)	1	0–4

The following morning (day 2), the cat was transferred to the critical care service. Systolic blood pressure was 80 mm Hg, and rectal temperature was 32.9°C (38.1–39.2°C).^8^ Repeat bloodwork revealed persistent hyperglycemia with a sBG of 23.87 mmol/L (430 mg/dl; RI [3.72–9.32 mmol/L]).^10^ Serum ketones were negative (Precision Xtra, Abbott Animal Health). A vBG showed a worsening metabolic acidosis with improving hyperlactatemia (Table 1). A CBC (Procyte Dx Hematology Analyzer, IDEXX Laboratories Inc) showed a non‐regenerative anemia and neutrophilia (Table 2). One unit (1 U; 0.25 U/kg) of regular insulin (HumulinR, Lilly USA LLC) was administered IV. Spot blood glucose one‐hour post‐insulin was 20.7 mmol/L (372 mg/dl; RI [3.72–9.32 mmol/L]),^10^ and the dose was repeated. Two additional doses (90 mg/kg and 150 mg/kg respectively) of calcium gluconate 10% diluted 1:1 with NaCl 0.9% were administered IV. Bradycardia worsened to 90 beats per minute and a single dose of atropine sulfate (Atropine Sulfate Injection 1/120 Grain, Med‐Pharmex) 0.04 mg/kg IV was administered with no effect. A calcium gluconate 10% continuous rate infusion (CRI) was initiated at 0.5 mls/kg/h. Bradycardia persisted and an electrocardiogram (cECG; MD9000vet, Meditech Equipment Co., Ltd) identified intermittent absence of P‐waves. A point‐of‐care thoracic ultrasound showed scant pleural effusion and markedly decreased cardiac contractility. Intravenous fluid and calcium gluconate therapies were discontinued, and a dobutamine CRI (2.5–3.75 mcg/kg/min; Dobutamine Injection USP, Hospira Inc.) was initiated. Intralipid emulsion therapy (ILT; Intralipid 20% Baxter Healthcare Corporation) was administered: 1.5 mls/kg IV bolus followed by 0.25mls/kg/min for one hour. Hypothermia worsened to 33.2°C (38.1–39.2°C)^8^ despite heat support and the cat's mentation deteriorated to dull. Serial vBGs showed a worsening metabolic acidosis and hypokalemia (Table 1). Crystalloid fluid therapy was resumed at a rate of 5mls/h with potassium supplementation (potassium chloride injection 2 mEq/ml, Hospira Inc Lake Forest, IL) at 80 mEq/L. High‐dose insulin/euglycemia therapy (HIET) was initiated: 1 U/kg IV bolus of regular insulin and a 1 g/kg IV bolus of dextrose (50% dextrose injection, USP 25 g/50 ml Hospira Inc.) were administered followed by a regular insulin CRI at 1 U/kg/h. Serum BG prior to HIET was 7.3 mmol/L (132mg/dl; RI [3.72–9.32mmol/L]).^10^ The insulin rate was decreased after the first hour to 0.5 U/kg/h. Intravenous crystalloid therapy with 5% dextrose was continued at 5 mls/h in conjunction with the insulin CRI. Serum BG was monitored every 30–60 min due to patient size and volume of pre‐sample required in a hemodynamically unstable patient. Within 5–10 min of starting HIET, there was a notable improvement in the patient's blood pressure, heart rate and temperature. After 2 h of therapy, blood pressure was 112 mm Hg, heart rate was 140 beats per minute with normal sinus rhythm, and temperature was 36.7°C. Serum potassium increased (Table 1) and potassium supplementation was decreased from 80 to 60 mEq/L. Hypoglycemia (BG less than 3.72 mmol/L) was identified five times during HIET; and was treated with intermittent dextrose boluses (0.5 g/kg IV) and an increase in dextrose supplementation from 5% to 7.5% with no change to the fluid rate of 5mls/h. Insulin therapy was discontinued after approximately three hours due to persistent hypoglycemia. At that time, the cat was ambulatory and interactive. Point‐of‐care ultrasound showed a marked improvement in cardiac contractility. Overnight dextrose supplementation was decreased then discontinued as euglycemia was maintained. Crystalloid fluids were continued at 5 mls/h with 60 mEq KCl/L supplementation. Temperature normalized and heat support were discontinued. Heart rate remained greater than 150 beats per minute and blood pressure remained between 90 mm Hg and 132 mm Hg on a dobutamine CRI at 3.75 mcg/kg/min.

Early the next day (Day 3), the cat became hypotensive with a decrease in SBP to 80 mmHg. A CRI of norepinephrine (Levophed TM, Norepinephrine Bitartrate Injection USP, Hospira Inc.) was initiated at 0.1 mcg/kg/min and titrated up to 0.2 mcg/kg/min. Mild respiratory effort was noted, and oxygen therapy at FiO2 0.4 (Snyder MFG.CO Intensive Care Unit) was provided. Point‐of‐care ultrasound revealed an increase in the volume of pleural effusion. Based on progression of clinical signs and financial constraints, the owners elected humane euthanasia.

## 3 DISCUSSION

This is the first case report describing hyperinsulinemia/euglycemia therapy for the treatment of amlodipine toxicosis in a cat. Calcium‐channel blockers are rapidly and completely absorbed after ingestion.^10^ Amlodipine has the highest bioavailability and volume of distribution in its class,^11^ is highly protein bound and hepatically metabolized prior to renal excretion. The elimination half‐life is potentially the longest of any CCB at 30 h in the dog, with a range of 30–60 h reported in the cat.^1^ Time to peak plasma concentration is not known in cats, but is reported as 6 h in dogs, and 6–12 h in humans.^1^ The minimum toxic dose has not been established in humans or animals; however, signs of toxicosis at therapeutic doses have been noted in all classes of CCB.^1^ Amlodipine toxicosis is the most commonly reported dihydropyridine toxicosis in animals, accounting for 83.2% of all dihydropyridine cases reported to the ASPCA APCC from 2000 to 2017.^1^ The clearance of CCBs is likely prolonged in disease states such as congestive heart failure, cardiomyopathy, or hypotensive overdose, which result in altered hepatic perfusion.^12^ Standard dosage is typically 0.625–1.25 mg per cat.^8^ Little is known about the cumulative effects of amlodipine, but signs of toxicosis in people may persist for up to 10 days, with mortality in symptomatic cases as high as 21%.^13^

Decontamination and limitation of toxin absorption is the first line of treatment if ingestion occurred within two hours of presentation.^1^ Emesis was not attempted in the cat reported here due to the chronicity of the exposure. Activated charcoal is recommended for the treatment of amlodipine toxicity to reduce gastrointestinal absorption of CCBs^1^; however, effectiveness of therapy depends on both the formulation and the time between ingestion and initiation of charcoal treatment.^1^ Charcoal was not administered in this case due to the concern for aspiration and the time elapsed since the last dose of amlodipine (approximately 16 h). A warm water enema could have been performed to facilitate evacuation of intestinal contents.^1^ Calcium gluconate is indicated to overcome inhibited calcium channels and promote inotropy, but response to treatment is inconsistent in severe intoxications.^5^ An exact dose of calcium is not described but a CRI titratable to a desirable blood pressure and heart rate has been suggested.^10^ The administration of calcium gluconate in this case led to ionized hypercalcemia without any clear benefit. Atropine may increase heart rate and improve cardiac output, however the effect is often transient.^2^ Furthermore, the resulting increase in myocardial oxygen demand can lead to myocardial injury when systemic and coronary hypotension are present.^14^ Atropine had no positive effect in the present case. Vasopressors can increase SBP by overriding the vasodilatory effects of amlodipine, but the resulting increase in SVR can lead to decreased cardiac output and altered perfusion of vascular beds.^5^ Treatment with adrenergic drugs may yield a poor response in patients with moderate to severe overdoses.^10^ Glucagon is a pancreatic peptide and cardiac inotrope.^1^ It increases cardiac output, lowers pulmonary arterial pressure, and pulmonary vascular resistance without any systemic vascular effects.^15^ Several animal models evaluating glucagon for the treatment of CCB toxicosis have shown an increase in HR and cardiac output, as well as AV block reversal; however, no effect on MAP or survival was observed.^10^ Glucagon was not used in the case reported here due to lack of availability and associated cost.

The use of intravenous lipid emulsion therapy (ILT) has been described for the treatment of various toxic substances with a high lipid solubility,^16^ including CCBs.^7^ There is however, a paucity of data regarding timing, dosing, and association with outcome when used for CCBs.^29^, ^31^ The mechanism of action is incompletely understood. One theory is that lipids sequester the lipophilic drug in a “lipid sink” reducing availability and promoting clearance through metabolism of drug‐containing chylomicrons.^16^ Another proposed benefit of ILT in cardiotoxic drugs is that increased availability of free fatty acids may prevent the myocardium from switching to glucose as its preferred energy substrate.^16^ Furthermore, long chain fatty acids may activate myocyte calcium channels resulting in increased calcium influx.^16^ The inhibition of amlodipine‐induced cardiomyoblast apoptosis using ILT has been demonstrated in a murine model.^17^ Improvement of clinical signs is reported within 20 min of administration^30^; however, response to therapy is unreliable.^29^ In the present case, the authors do not believe that the patient exhibited any clinical improvement following the implementation of ILT, which prompted the initiation of HIET. Complications associated with ILT are infrequent but include microbial or particulate contamination of the lipid product, pancreatitis, hyperlipidemia, or an allergic‐type reaction to the infusion.^16^

There are three major mechanisms by which HIET is believed to improve survival following CCB overdose: (1) increased inotropy, (2) increased intracellular glucose transport, and (3) vasodilation.^5^ High‐dose insulin increases coronary blood flow without increasing oxygen demands, contrary to catecholamines.^6^ Insulin also increases eNOS activity leading to systemic, coronary, and pulmonary vasodilation.^5^ Cardiogenic shock is characterized by altered microvascular perfusion and a failure to supply capillary beds and the surrounding tissues with metabolic substrate. By increasing eNOS activity, insulin enhances microvascular hemodynamics improving tissue perfusion, decreasing afterload, and increasing cardiac output.^5^ In cell cultures, supraphysiologic doses of insulin are required to increase eNOS activity above basal concentrations, which may explain why hyperinsulinemia is needed to elicit these beneficial vascular effects.^5^

A study by Kline et al compared normal saline, epinephrine, glucagon, calcium chloride and HIET for the treatment of verapamil toxicosis in dogs. Dogs that received HIET had an increased survival rate, improved maximum elastance, left ventricular end diastolic pressure, ventricular relaxation, and coronary artery blood flow.^18^ Beta‐blocker overdose is often used in experimental models to extrapolate data which can then be applied to clinical CCB and beta‐blocker toxicities as both drug classes result in blockade of calcium influx through the L‐type calcium channels.^20^ An experimental study by Kerns et al showed that insulin‐treated anesthetized dogs following propranolol‐induced cardiomyopathy had improved cardiodynamics and hemodynamics, as well as increased myocardial glucose uptake compared to dogs treated with glucagon or epinephrine. Survival was significantly higher in the insulin group compared with the glucagon and epinephrine groups.^15^ Krukenkamp et al. induced myocardial depression in dogs using propranolol and found 80% reversal following HIET, as well as a statistically significant increase in peak blood pressure without changing myocardial oxygen consumption.^19^ Two experimental porcine models evaluated the use of vasopressors and HIET in propranolol‐induced cardiogenic shock.^21^, ^22^ The first study found that pigs treated with insulin and dextrose had a consistent improvement in cardiac output over the duration of treatment compared with the pigs treated with vasopressors alone, which had a progressive worsening of cardiac output.^21^ The authors concluded that higher insulin doses may be required in the presence of vasopressors to overcome increased SVR and decreased cardiac output.^21^ The second study found that patients treated with norepinephrine after maximizing high‐dose insulin therapy had improved brain tissue oxygenation compared to HIET alone^22^ and that patients with profound hypotension (<55 mm Hg) could benefit from vasopressors after maximizing HIET.

There is strong evidence supporting the benefits of HIET in humans with CCB toxicosis; however, there is little clinical evidence in the veterinary literature to support its use. One case report by Maton et al describes the use of HIET in conjunction with ILT to treat diltiazem toxicosis in a dog. In that case, HIET was initiated after clinical signs did not improve despite decontamination, calcium gluconate, dopamine CRI, glucagon, insulin pulse therapy with dextrose supplementation at 5%, and ILT. Dextrose supplementation was increased to 10% at initiation of HIET and a regular insulin CRI was initiated at 1 U/kg/h. Within one hour of starting the insulin CRI clinical signs improved. The dog received a total of 20 hours of ILT therapy and 17 hours of insulin CRI and dextrose CRI which were slowly weaned. The patient was discharged without long‐term complications.^7^ In the present case, a notable response to HIET following ILT was appreciated as evidenced by an increase in temperature, heart rate, blood pressure and contractility within 15 min of initiating therapy.

A contributing factor in the decision to euthanize was the worsening pleural effusion and development of respiratory effort. Pleural effusion in cats has been linked to several disease processes with cardiac dysfunction reported as the most common etiology.^26^ Fluid overload is a life‐threatening complication of fluid therapy in hospitalized patients which can manifest as pleural effusion.^27^, ^28^ The direct effects of CCB on pulmonary vasomotor tone resulting in respiratory deterioration should also be considered. Both non‐cardiogenic pulmonary edema (NCPE) and pleural effusion have been identified as complications of severe CCB toxicity in humans, occurring in 64% and 42% respectively of patients with confirmed amlodipine toxicosis requiring vasopressors and inotropic support.^23^ The presumed pathophysiology of NCPE in CCB toxicosis is selective precapillary vasodilation and suppression of autoregulatory responses in the lungs. Consequently, any increase in cardiac output may lead to increased pulmonary hydrostatic pressure and worsening pulmonary infiltrates.^23^ The use of high osmolality intravenous fluids may also contribute to increased intravascular volume and worsening NCPE. In the absence of thoracic radiographs, it is not known whether pulmonary edema was a contributing factor to this cat's respiratory compromise.

## 4 CONCLUSION

Both HIET and ILT are cost‐effective, readily available therapies that can be implemented for severe CCB toxicity in cats. The dosing recommendation for animals is a 1 U/kg insulin bolus followed by a 1 U/kg/h CRI for 1 hour, followed by 0.5 U/kg/h until resolution of clinical signs.^10^ There is no set protocol for timing of discontinuation of HIET, however the goal of therapy is hemodynamic stability. Based on previous reports and human guidelines, prolonged therapy may be needed. The primary complications associated with HIET are hypoglycemia and hypokalemia; both of which can be anticipated, monitored, and addressed. The initiation of vasopressor therapy in the context of amlodipine toxicosis should be approached with caution and considered after initiation and optimization of HIET. The authors also recommend monitoring of magnesium and phosphorus, which have a complex interplay with insulin and are vital for myocardial cellular activity. The wide availability of insulin and dextrose, low expense, and minimal adverse event profile make HIET an attractive therapeutic option for CCB toxicosis. Although the outcome was unsatisfactory in this case, the authors feel that the response to HIET supports the use of this therapy in cats with CCB toxicosis.

## CONFLICT OF INTEREST

The authors declare no conflicts of interest.

## AUTHOR CONTRIBUTIONS

A Tinsman: conducted a literature search on the topic, drafted and revised the paper. TJ Bellis: conducted a literature search on the topic and revised the paper. Both parties were responsible for patient management and treatment while in hospital.

## ETHICAL APPROVAL

Ethical approval is not required at our institution to publish an anonymous case report.

## Data Availability

Data sharing is not applicable to this article as no datasets were generated or analyzed during the current study.
